# Mutational analysis of COL1A1 and COL1A2 genes among Estonian osteogenesis imperfecta patients

**DOI:** 10.1186/s40246-017-0115-5

**Published:** 2017-08-15

**Authors:** Lidiia Zhytnik, Katre Maasalu, Ene Reimann, Ele Prans, Sulev Kõks, Aare Märtson

**Affiliations:** 1 Department of Traumatology and Orthopedics, University of Tartu, Puusepa 8, 51014 Tartu, Estonia; 2 Clinic of Traumatology and Orthopedics, Tartu University Hospital, Puusepa 8, 51014 Tartu, Estonia; 3 Centre of Translational Medicine, University of Tartu, Ravila 14a, 50411 Tartu, Estonia; 4 Department of Pathophysiology, University of Tartu, Ravila 19, 50411 Tartu, Estonia

**Keywords:** Osteogenesis Imperfecta, Collagen I, *COL1A1*, *COL1A2*, Sanger sequencing

## Abstract

**Background:**

Osteogenesis imperfecta (OI) is a rare bone disorder. In 90% of cases, OI is caused by mutations in the *COL1A1/2* genes, which code procollagen α1 and α2 chains. The main aim of the current research was to identify the mutational spectrum of *COL1A1/2* genes in Estonian patients. The small population size of Estonia provides a unique chance to explore the collagen I mutational profile of 100% of OI families in the country.

**Methods:**

We performed mutational analysis of peripheral blood gDNA of 30 unrelated Estonian OI patients using Sanger sequencing of *COL1A1* and *COL1A2* genes, including all intron-exon junctions and 5′UTR and 3′UTR regions, to identify causative OI mutations.

**Results:**

We identified *COL1A1/2* mutations in 86.67% of patients (26/30). 76.92% of discovered mutations were located in the *COL1A1* (*n* = 20) and 23.08% in the *COL1A2* (*n* = 6) gene. Half of the *COL1A1/2* mutations appeared to be novel. The percentage of quantitative *COL1A1/2* mutations was 69.23%. Glycine substitution with serine was the most prevalent among missense mutations. All qualitative mutations were situated in the chain domain of pro-α1/2 chains.

**Conclusion:**

Our study shows that among the Estonian OI population, the range of collagen I mutations is quite high, which agrees with other described OI cohorts of Northern Europe. The Estonian OI cohort differs due to the high number of quantitative variants and simple missense variants, which are mostly Gly to Ser substitutions and do not extend the chain domain of *COL1A1/2* products.

## Background

Despite being a rare genetic bone fragility disorder, osteogenesis imperfecta (OI) is among the most widely occurring of rare congenital skeletal dysplasias [1]. OI prevalence is estimated 1/10,000–20,000 at birth [2, 3]. OI is characterized by low bone mineral density, recurrent fractures, skeletal deformations, and blue eye sclera [2, 4–6]. Other remarkable features of OI include Dentinogenesis Imperfecta, triangular face, hearing loss, joint laxity, short stature, and easy bruising [2, 4–6].

OI has many manifestations and is considered a group of disorders. Phenotypes range from mild osteopenia to severe deformities or even mortality. In 1979, Sillence described four OI types (I–IV) according to phenotype severity [5]. Recent updated classification distinguishes three additional types with specific histologies (V–VII) [4, 7]. Genetic OI classification considers every OI gene as a separate OI type and so far includes OI types I–XVII [4, 8, 9].

The genetics of the disorder reflect the complexity of the OI phenotype range. Up to 21 different genes have been associated with occurrence of OI [10–19]. Previous studies have shown that the primary cause of OI are mutations in the *COL1A1/2* genes, which code procollagen type I α1 and α2 chains, respectively [20]. Despite the approximately 1500 mutations already described in collagen type I genes, investigators continue to report novel mutations [21]. Moreover, there is still some controversy regarding the proportion of collagen mutations reported in different populations, which have ranged from 60 to 95% [9, 10]. In this context, we believe that population-based studies of OI genetics might broaden current knowledge of collagen I mutations and OI.

Due to Estonia’s small population (1.3 million) and centered treatment, follow-up, and research of all OI patients at the OI Center of the Traumatology and Orthopedics Clinic, Tartu University (TU) Hospital, it was possible to perform analysis of *COL1A1/2* mutations among the whole Estonian OI population [22]. Herein, we describe for the first time the mutational spectrum of *COL1A1/2* genes among 30 unrelated OI patients, from 30 Estonian OI families, which we estimate to constitute ~ 100% of OI cases in Estonia.

## Methods

### Subjects

The patients included in the study are treated and followed-up by the OI Center of the Traumatology and Orthopedics Clinic, TU Hospital.

A total of 30 OI patients from 30 unrelated families were included in the study. Data regarding the OI types of the subjects were obtained from the medical records of TU Hospital [23]. All new OI cases across Estonia are registered by and treated at TU Hospital’s OI Center. Thus, it can be estimated that as of May 2017, the current patient cohort represented ~ 100% of the Estonian OI population.

No patient came from a consanguineous family. Mutational analysis of the *COL1A1/2* genes was performed on a younger affected member of every OI family included in the study.

In accordance with the Declaration of Helsinki, all patients or their legal representatives signed an informed consent form prior to participation. The study was approved by the University of Tartu’s Ethical Review Committee on Human Research (permit no. 221/M-34).

### Genealogical description

Genealogical data of OI history in the family, consanguinity, and miscarriages was obtained from each patient or their representative. We constructed pedigree trees per kindred using the “Kinship2” package in R v3.3.2 [24].

### Mutational analysis of the *COL1A1/2* genes

Genomic DNA (gDNA) was purified from 3 ml of ethylenediaminetetraacetic acid (EDTA) preserved whole blood samples—stored at −80 °C—using a Gentra Puregene Blood Kit (Quiagen, Germany) following the manufacturer’s protocol.

PCR amplification and Sanger sequencing were performed as described previously [25]. Sequence products were analyzed using Applied Biosystems’ Sequence Scanner v1.0 and Mutation Surveyor DNA Variant analysis software v5.0.1. (Softgenetics, USA) and aligned to the GenBank human reference genome sequences of *COL1A1* (gDNA NG_007400.1, complementary (cDNA) NM_000088.3), and *COL1A2* (gDNA NG_007405.1, cDNA NM_000089.3). Raw sequencing data are available from the authors upon request. We focused on non-synonymous and splice-site variants absent from the publicly available normal datasets (including dbSNP135 and the 1000 Genomes Project) [26, 27]. We used the PolyPhen-2, SIFT, and MutationTaster software tools to predict the functional effects and pathogenicity of mutations [28–30]. Variants absent from the osteogenesis imperfecta mutation database were considered novel (http://www.le.ac.uk/ge/collagen/) [21, 31].

All statistical analyses were carried out with R v3.3.2. software (R Team, Austria) [32]. To assess the distribution of *COL1A1/2* mutations and compare them to other studied OI populations, percentage differences were used.

## Results

**Fig. 1:**
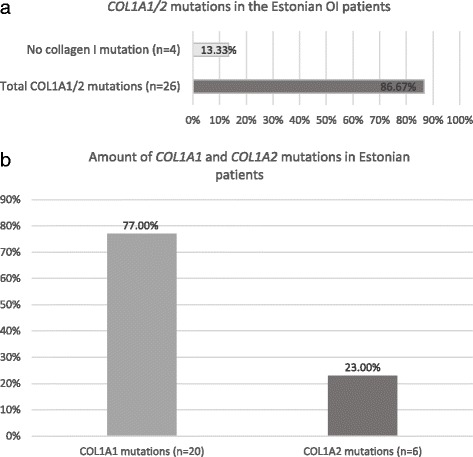
Diagram of collagen type I mutation distribution Estonian OI patients. **a** Percentage of patients with *COL1A1/2* mutations vs patients negative for collagen I mutations. **b** Percentage of mutations in the *COL1A1* and *COL1A2* genes

Mutational analysis of the *COL1A1/2* genes of Estonian OI patients highlighted OI causative mutations in 26 of 30 patients (86.67%) (Fig. 1a). The number of patients harboring *COL1A1* mutations was 20 (76.92%); *COL1A2* mutations were found in 6 patients (23.08%) (Fig. 1b). A list of the mutations and their characteristics can be found in Table 1.

**Table 1:** Mutational spectrum of the *COL1A1* and the *COL1A2* genes among Estonian OI patients

No	Patient ID	Gene	Mutation	Exon	Mutation type	Protein alteration	Sillence OI type
1	EE01#	COL1A2	c.1630G>GT*	Exon 28	Missense	p.Gly544Cys	III
2	EE02	COL1A1	c.1821 + 1G>GA	Intron 26	Splice site	–	III
3	EE03	COL1A1	c.1897G>GT*	Exon 26	Nonsense	p.Glu633*	IV
4	EE04	COL1A1	c.750 + 2T>TA*	Intron 10	Splice site	–	IV
5	EE05	COL1A1	c.1821 + 1G>GA	Intron 26	Splice site	–	I
6	EE07#	COL1A1	c.2317G>T*	Exon 33_34	Missense	p.Gly773Cys	II
7	EE08	COL1A1	c.3217G>GA*	Exon 45	Missense	p.Gly1073Ser	III
8	EE09	COL1A1	c.1155 + 2T>TG*	Intron 17	Splice site	–	I
9	EE10	COL1A1	c.1128_hetdelT	Exon 17	Frameshift	p.Gly377Alafs*164	I
10	EE11#	COL1A1	c.3235G>GA	Exon 45	Missense	p.Gly1079Ser	I
11	EE13	COL1A1	c.2089C>CT	Exon 31	Nonsense	p.Arg697*	IV
12	EE14#	COL1A1	c.904-9G>GA	Intron 13	Splice site	–	I
13	EE15	COL1A2	c.1009G>GA	Exon 19	Missense	p.Gly337Ser	III
14	EE16#	COL1A2	c.2324G>GA	Exon 38	Missense	p.Gly775Glu	III
15	EE17#	COL1A1	c.3045 + 1G>GA	Intron 42	Splice site	–	IV
16	EE18	COL1A1	c.505G>GA*	Exon 6	Missense	p.Glu169Lys	I
17	EE19	COL1A1	c.299-1G>GC*	Intron 3	Splice site	–	IV
18	EE20	COL1A2	с.937-3С>CT	Intron 18	Splice site	–	I
19	EE21	COL1A1	c.3262G>GT*	Exon 46	Nonsense	p.Gly1088*	IV
20	EE22	COL1A1	c.3262G>GT*	Exon 46	Nonsense	p.Gly1088*	I
21	EE24	COL1A1	c.1767 + 5G>GA*	Intron 25	Splice site	–	IV
22	EE25	COL1A1	c.1354-2A>AG	Intron 20	Splice site	–	I
23	EE27#	COL1A1	c.3208-1G>GA*	Intron 44	Splice site	–	I
24	EE29#	COL1A2	c.865G>AG	Exon 17	Missense	p.Gly289Ser	III
25	EE30	COL1A2	c.2026-1_2031het dup*	Intron-Exon 34	Splice site, frameshift	–	III/IV
26	EE31#	COL1A1	c.1081C>CT	Exon 17	Nonsense	p.Arg361*	I

Patients with de novo mutations and without OI history in the family are marked with an octothorp (#). Novel mutations unreported in the collagen type I variant database (http://www.le.ac.uk/ge/collagen/) are marked with an asterisk (*). In cases of heterozygous mutation, both the wild type and the mutated allele are indicated after an arrow (>)

The number of novel mutations was 13/26 (50%) (Table 1). Half of the *COL1A1* and *COL1A2* mutations appeared to be undescribed in the collagen type I mutation database. Patient EE26 had a heterozygous non-synonymous rs1800215 SNP (p.Ala1075Thr) in the *COL1A1* gene, which was described before as a benign variant (data not shown). [33]

Twenty-five mutations had an autosomal dominant inheritance pattern (Table 1). Of these, eight patients had no previous history of OI in the family. Thus, we assumed that their parents and relatives, who did not have any clinical features of OI, are not carriers of these mutations.

Patient EE07 had a recessive missense mutation. Mutational analysis showed that their parents are not carriers of the mutation, which confirmed the de novo nature of the mutation.

**Fig. 2:**
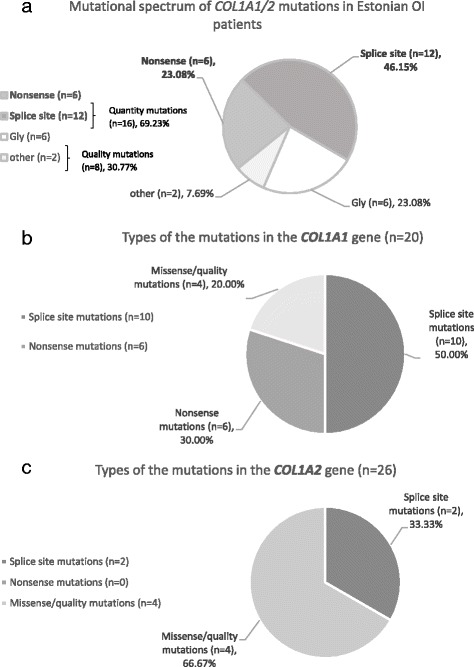
**a** Mutational spectrum of *COL1A1/2* mutations in Estonian OI patients. Distribution of the *COL1A1* (**b**) and *COL1A2* (**c**) mutations according to mutation type

We found 12/26 mutations (46.15%) had altering splice sites, 10 and 2 in the *COL1A1* and *COL1A2* genes, respectively. One of the patients harbored a deletion capturing both coding and intronic sequence, in exon-intron 34 (EE30). Nonsense mutations were present in 6 patients (23.08%), all in the *COL1A1* gene. Overall, quantitative mutations were present in 18 patients (16 in *COL1A1* and 2 in *COL1A2* genes) (Fig. 2).

Missense mutations, associated with collagen I quality defects, were indicated in eight patients (30.77%), four in *COL1A1* and four in *COL1A2* genes. Of these, seven were Glycine substitutions (four of the *COL1A2* and three of the *COL1A1* missense mutations). In four cases, Glycine was substituted with Serine, two in the *COL1A1* and *COL1A2* genes, respectively (Fig. 2).

A c.3262G>T (*COL1A1*) mutation was detected in two patients (EE21 and EE22), who were thought to be unrelated (Table 1). Investigation of the pedigree trees revealed a distant relationship between the families four generations back, of which the patients were not aware. Two identical splice site mutations at c.1821 + 1G>A (*COL1A1*) in intron 26 were identified in patients EE02 (type III OI) and EE05 (type I OI) (Table 1). This mutation arose independently in the patients and caused phenotypes of different severity.

## Discussion

Collagen I mutations were found in 26/30 (87%) studied OI patients. Previous findings have suggested collagen mutations ranging from 60 to 90% among different OI populations and study cohorts [25]. In a Finnish OI study, 90.7% of patients harbored collagen I mutations [34], which is higher than we found among Estonian OI patients. In Pollitt et al.’s study, collagen I mutations were revealed in 75% of OI patients [35], which is slightly lower than our Estonian cohort. Our data is in good agreement with research on the genetic epidemiology of the Swedish OI population, of which 87% had collagen I mutations [36]. The results of our study are also in concordance with Bardai et al.’s recent study of a large number (598) of OI individuals, where collagen type I mutations were found in 86% of OI patients of all types and different ethnic groups [19].

In some population studies, the amount of collagen I mutations were also lower. For example, in 51.4% of Taiwanese patients (*N* = 72), 52.2% of Korean patients (*N* = 67), and 59.4% of Vietnamese OI patients (*N* = 91) [25, 37, 38]. Due to the difficulties in arranging large cross-population studies of a rare disorder in populous countries, results can often be fragmented, which complicates population-wide estimates [39, 40]. However, questions about the lower collagen type I mutational pattern of OI patients from Asian populations remain.

The proportions of *COL1A1* and *COL1A2* in Estonian, Finnish, and Swedish OI populations were surprisingly similar, 77 and 23%, 78 and 22%, and 79 and 21%, respectively [34, 36]. Similar values were reported by Pollitt et al., where 77% of mutations occurred in the *COL1A1* and 23% in the *COL1A2* gene (*N* = 83) [35]. In Bardai et al.’s 2016 study, 69% were *COL1A1* and 31% *COL1A2* mutations, which is similar to the beforementioned results [19].

The Estonian cohort also has a high proportion of quantitative mutations compared to qualitative collagen mutations, 69 and 31%, respectively. In the Finnish OI cohort, 67% of mutations were quantitative and 33% qualitative [34]. In the work of Pollitt et al., 35% of mutations were qualitative and 65% quantitative [35]. In the Swedish population, the proportions were almost equal (53 and 47%) [36]. Interestingly, we found only two quantitative mutations in the *COL1A2* gene, which matches previous reports about comparatively lower numbers of quantitative mutations of this gene [34–36]. Due to the higher number of mutations leading to haploinsufficiency in the *COL1A1* gene compared to the *COL1A2* gene, patients harboring mutations in the *COL1A1* gene had milder phenotypes (I, IV) compared to patients with *COL1A2* mutations (type III, except EE20 who had a splice site mutation and OI type I).

Glycine substitutions composed the vast majority of missense mutations (7 of 8 cases), with serine being the most substituted amino acid (4 of 7 cases), which supports previous findings. Curiously, all missense mutations were situated in triple helical chain domains (aa residues 162–1218 α1; aa residues 80–1102 α2) of *COL1A1/2* gene products. Only one mutation (patient EE07 with OI type II) altered the “lethal cluster” proposed by Marini et al. [41].

Half of the mutations (50%) we found appeared to be novel. Despite the numerous works on collagen I mutations and a growing list of identified mutations, the number of revealed novel variants was high, which underlines the individual nature of OI mutations [19, 35, 36]. Half of the glycine substitutions (4 of 7) were even absent from the collagen I mutational database.

Despite sharing of the same mutation, patients may develop different phenotypes, as in the case of patients EE02 and EE05, who had type III and I OI, respectively. Genotype-phenotype correlations remain an unresolved issue in our understanding of OI. Cases of inter- and intra-familial OI diversity are not rare. Not only genetics, but additional factors, such as epigenetics and environment might contribute to the development of specific OI phenotypes. This leads to many questions and the need to further investigate potential OI factors.

Sanger sequencing is a powerful and accurate method of mutational analysis and allows the identification of frameshift, and missense and nonsense mutations in the coding regions of genes. Moreover, due to the special design of the primers distant from intron-exon junction regions, we could asses splice site mutations of the *COL1A1/2* genes, which are the cause of quantitative collagen defects. However, the current study had some limitations. We could not identify whole gene or exon deletions and duplications, which could have slightly reduced the number of discovered *COL1A1/2* mutations. In addition, due to the small population size of Estonia, our cohort was limited. We cannot exclude the possibility that the small sample size might be the cause of differences compared to the results of other studies.

## Conclusion

This paper has described the mutational spectrum of *COL1A1/2* genes among 30 Estonian OI patients, which were estimated to represent ~ 100% of OI families in Estonia at the time. We identified collagen I mutations in 87% of Estonian OI families. The number of quantitative mutations (69%) was high compared to other European OI cohorts. All missense mutations of our Estonian patients altered the triple helical chain domain of α1 and α2 procollagen chains. One mutation was situated in the lethal cluster. A normal distribution of novel collagen mutations (50%) among the *COL1A1* (77%) and *COL1A2* (23%) genes, and mostly glycine substitutions were observed, compared to other OI cohorts of Northern Europe. Four patients that showed no collagen type I mutations will be further studied using whole exome sequencing analysis to identify disease causing variants.

## Abbreviations

3’UTR: Three prime untranslated region
5’UTR: Five prime untranslated region
cDNA: Complementary DNA
EDTA: Ethylenediaminetetraacetic acid
gDNA: Genomic DNA
OI: Osteogenesis Imperfecta
TU: University of Tartu
